# Supplementary material for: Patterns of HIV-1 viral load suppression and drug resistance during the dolutegravir transition: a population-based longitudinal study
Source: medRxiv. 2025 Sep 2:2025.09.01.25334862. Preprint. [Version 1] doi: 10.1101/2025.09.01.25334862 (PMC12424902; doi:10.1101/2025.09.01.25334862)
Supplement: Supplement 1 [file media-1.pdf]

Patterns of HIV-1 viral load suppression and drug resistance during the dolutegravir transition: a population-based longitudinal study: Supplementary Tables

Supplementary Table 1: Rakai Community Cohort Study survey dates

Round	Survey start date	Survey mid date	Survey end date
1	1994-11-05	1995-03-20	1995-07-18
2	1995-01-12	1996-01-10	1996-05-01
3	1996-01-12	1996-11-20	1997-04-23
4	1997-06-06	1997-11-21	1998-05-12
5	1997-08-05	1998-08-06	1999-03-15
6	1999-04-06	1999-09-07	2000-02-09
7	2000-03-20	2000-08-08	2001-02-19
8	2001-04-03	2001-10-23	2002-05-31
9	2002-07-15	2003-01-28	2003-08-01
10	2003-09-26	2004-04-23	2004-11-23
11	2005-01-13	2005-10-13	2006-06-30
12	2006-03-31	2007-06-28	2008-04-24
13	2008-06-17	2009-03-17	2009-12-04
14	2010-01-18	2010-09-24	2`011-06-21
15	2011-08-10	2012-04-02	2013-05-29
16	2013-07-08	2014-04-07	2015-01-28
17	2015-02-23	2015-11-03	2016-09-02
18	2016-10-03	2017-07-17	2018-05-21
19	2018-06-19	2019-06-20	2020-11-04
20	2021-02-01	2022-03-04	2023-03-07

Supplementary Table 2: Antiretroviral therapy to which resistance was predicted

Generic name	Abbreviation	Class
bictegravir	BIC	INSTI
cabotegravir	CAB	INSTI
dolutegravir	DTG	INSTI
elvitegravir	EVG	INSTI
raltegravir	RAL	INSTI
doravirine	DOR	NNRTI
efavirenz	EFV	NNRTI
Etravirine	ETR	NNRTI
nevirapine	NVP	NNRTI
rilpivirine	RPV	NNRTI
abacavir	ABC	NRTI
didanosine	DDI	NRTI
emtricitabine	FTC	NRTI
lamivudine	3TC	NRTI
stavudine	D4T	NRTI
tenofovir	TDF	NRTI
zidovudine	AZT	NRTI
atazanavir	ATV	PI
darunavir	DRV	PI
fosamprenavir	FPV	PI
indinavir	IDV	PI
lopinavir	LPV	PI
nelfinavir	NFV	PI
saquinavir	SQV	PI
tipranavir	TPV	PI

Supplementary Table 3: Recommended ART regimen among RCCS participants by year

Year	First line regimen	Second line regimen
2012	**Preferred**	**Preferred**
	TDF/3TC + NVP	AZT + 3TC + ATV/r
	TDF/3TC + EFV	AZT + 3TC + LPV/r
	**Alternative**	**Alternative**
	AZT/3TC + NVP	TDF + 3TC + ATV/r
	AZT/3TC + EFV	TDF + 3TC + LPV/r
2016	**Preferred**	**Preferred**
	TDF+3TC+EFV	2 NRTIs and ritonavir-boosted atazanavir (ATV/r)
		e.g.
	**Alternative (only if EFV is contraindicated)**	After failing on TDF + 3TC or ABC+3TC based regimen: AZT+3TC / ATV/r
	TDF+3TC+DTG	
		After failing on AZT+3TC based regimen: TDF + 3TC/ ATV/r
2018	**Preferred**	**Preferred**
	TDF+3TC+DTG	AZT+3TC+ATV/r
	**Alternative**	**Alternative**
	TDF+3TC+EFV, ABC+3TC+DTG	AZT+3TC+LPV/r
		AZT+3TC+DTG
	**Adult women and adolescent girls of childbearing potential who are pregnant, intend to get pregnant or not on effective contraception**	
	TDF+3TC+EFV	
	**Alternative**	
	TDF+3TC+ATVr or ABC+3TC+EFV	
2022	**Preferred**	**Preferred**
	TDF + 3TC + DTG	AZT+3TC+ATV/r
		TDF+3TC+ATV/r
	**Alternatives**	
	TDF + 3TC + EFV400	**Alternative**
	ABC + 3TC +DTG	AZT+3TC+LPV/r
	ABC +3TC +EFV400	TDF+3TC+LPV/r
	TDF +3TC + ATV/r or ABC 3TC + ATV/r	

Supplementary Table 4: Demographics of RCCS participants by survey round.

		Survey Round					
		**2012**	**2014**	**2015**	**2017**	**2019**	**2022**
Overall		17,034	17,979	19,317	19,775	19,276	15,947
Age (median [IQR])		28 [13]	29 [15]	29 [14]	29 [16]	29 [17]	30 [18]
Age category							
	(14,24]	5,977 (35.1%)	6,404 (35.6%)	6,813 (35.3%)	6,968 (35.2%)	6,679 (34.6%)	5,468 (34.3%)
	(24,34]	6,394 (37.5%)	6,282 (34.9%)	6,411 (33.2%)	6,358 (32.2%)	5,913 (30.7%)	4,607 (28.9%)
	(34,49]	4,663 (27.4%)	5,293 (29.4%)	6,093 (31.5%)	6,449 (32.6%)	6,684 (34.7%)	5,872 (36.8%)
Community type							
	Agrarian	7,586 (44.5%)	7,947 (44.2%)	8,565 (44.3%)	8,488 (42.9%)	8,589 (44.6%)	5,747 (36%)
	Fishing	3,851 (22.6%)	3,923 (21.8%)	4,234 (21.9%)	4,748 (24%)	4,058 (21.1%)	4,343 (27.2%)
	Trading	5,597 (32.9%)	6,109 (34%)	6,518 (33.7%)	6,539 (33.1%)	6,629 (34.4%)	5,857 (36.7%)
Sex							
	F	9,194 (54%)	9,669 (53.8%)	10,446 (54.1%)	10,534 (53.3%)	10,415 (54%)	8,537 (53.5%)
	M	7,840 (46%)	8,310 (46.2%)	8,871 (45.9%)	9,241 (46.7%)	8,861 (46%)	7,410 (46.5%)

Percentages represent the proportion of total participant-visits in each survey round belonging to each sub-category.

Supplementary Table 5: Demographics of RCCS participants living with HIV by survey round.

		Survey Round					
		**2012**	**2014**	**2015**	**2017**	**2019**	**2022**
Overall		3,498	3,396	3,616	3,633	3,347	2,893
Age (median [IQR])		32 [10]	33 [12]	34 [11]	34 [11]	36 [12]	37 [11]
Age category							
	(14,24]	532 (15.2%)	481 (14.2%)	450 (12.4%)	366 (10.1%)	267 (8%)	199 (6.9%)
	(24,34]	1,663 (47.5%)	1,471 (43.3%)	1,483 (41%)	1,451 (39.9%)	1,211 (36.2%)	931 (32.2%)
	(34,49]	1,303 (37.2%)	1,444 (42.5%)	1,683 (46.5%)	1,816 (50%)	1,869 (55.8%)	1,763 (60.9%)
Community type							
	Agrarian	1,049 (30%)	1,043 (30.7%)	1,099 (30.4%)	1,031 (28.4%)	1,027 (30.7%)	650 (22.5%)
	Fishing	1,598 (45.7%)	1,522 (44.8%)	1,600 (44.2%)	1,737 (47.8%)	1,503 (44.9%)	1,534 (53%)
	Trading	851 (24.3%)	831 (24.5%)	917 (25.4%)	865 (23.8%)	817 (24.4%)	709 (24.5%)
Sex							
	F	2,171 (62.1%)	2,111 (62.2%)	2,297 (63.5%)	2,240 (61.7%)	2,142 (64%)	1,848 (63.9%)
	M	1,327 (37.9%)	1,285 (37.8%)	1,319 (36.5%)	1,393 (38.3%)	1,205 (36%)	1,045 (36.1%)

Percentages represent the proportion of total participant-visits in each survey round belonging to each sub-category.

Supplementary Table 6: Prevalence of HIV, treatment-experienced HIV, and viremic HIV among all RCCS participants.

		HIV				Treatment-experienced PLHIV					Viremic HIV				
**Survey round**	***n***	**Obs (%)**	**Prev. (95% CI)**	**Prev. ratio (95% CI)**	***p*-value**	**n**	**Obs (%)**	**Prev. (95% CI)**	**Prev. ratio (95% CI)**	***p*-value**	**n**	**Obs (%)**	**Prev. (95% CI)**	**Prev. ratio (95% CI)**	***p*-value**
2012	17,034	3,498 (20.5%)	20.5 (19.9, 21.2)	ref	ref	17,034	1,240 (7.3%)	7.3 (6.9, 7.7)	ref	ref					
2014	17,979	3,396 (18.9%)	18.9 (18.3, 19.5)	0.92 (0.89, 0.95)	<0.0001	17,979	2,120 (11.8%)	11.8 (11.3, 12.3)	1.62 (1.54, 1.7)	<0.0001	17,942	1,442 (8%)	8 (7.6, 8.4)	ref	ref
2015	19,317	3,616 (18.7%)	18.7 (18.2, 19.3)	0.91 (0.88, 0.94)	<0.0001	19,317	2,826 (14.6%)	14.6 (14.1, 15.1)	2.01 (1.91, 2.12)	<0.0001	19,311	1,030 (5.3%)	5.3 (5, 5.7)	0.66 (0.62, 0.71)	<0.0001
2017	19,775	3,633 (18.4%)	18.4 (17.8, 18.9)	0.89 (0.87, 0.92)	<0.0001	19,775	3,143 (15.9%)	15.9 (15.4, 16.4)	2.18 (2.07, 2.31)	<0.0001	19,765	726 (3.7%)	3.7 (3.4, 3.9)	0.46 (0.42, 0.5)	<0.0001
2019	19,276	3,347 (17.4%)	17.4 (16.8, 17.9)	0.85 (0.82, 0.88)	<0.0001	19,276	3,064 (15.9%)	15.9 (15.4, 16.4)	2.18 (2.06, 2.31)	<0.0001	19,262	453 (2.4%)	2.4 (2.1, 2.6)	0.29 (0.26, 0.32)	<0.0001
2022	15,947	2,893 (18.1%)	18.1 (17.6, 18.7)	0.88 (0.85, 0.92)	<0.0001	15,947	2,731 (17.1%)	17.1 (16.6, 17.7)	2.35 (2.22, 2.49)	<0.0001	15,917	278 (1.7%)	1.7 (1.6, 2)	0.22 (0.19, 0.25)	<0.0001

Viral load measurements were not routinely collected in the 2012 survey round and we are therefore unable to estimate the prevalence of suppression among people living with HIV (PLHIV) for this time frame. PLHIV with missing viral load data in later survey rounds were dropped when estimating the prevalence of suppressed HIV. Estimates were generated using Poisson regression with robust standard errors with survey round as a predictor variable. Generalized estimating equations with correlation structure selection by Quasi Information Criterion value (PLHIV, viremic PLHIV: independence) were used to account for repeat participants across study rounds. 95% confidence intervals indicate the Wald confidence interval around the mean value in each category. *p*-values that coefficients are different from 0 at the level were calculated using the Wald method.

Supplementary Table 7: Prevalence of treatment-experience and suppression among RCCS participants living with HIV.

		Treatment-experienced PLHIV				Suppressed PLHIV				
**Survey round**	***n***	**Obs (%)**	**Prev. (95% CI)**	**Prev. ratio (95% CI)**	***p*-value**	**n**	**Obs (%)**	**Prev. (95% CI)**	***Prev. ratio (95% CI)***	***p*-value**
2012	3,498	1,240 (35.4%)	35.4 (33.9, 37.1)	ref	ref					
2014	3,396	2,120 (62.4%)	62.4 (60.8, 64.1)	1.76 (1.68, 1.84)	<0.0001	3,359	1,917 (57.1%)	57.1 (55.4, 58.8)	ref	ref
2015	3,616	2,826 (78.2%)	78.2 (76.8, 79.5)	2.2 (2.11, 2.31)	<0.0001	3,610	2,580 (71.5%)	71.5 (70, 73)	1.25 (1.21, 1.29)	<0.0001
2017	3,633	3,143 (86.5%)	86.5 (85.4, 87.6)	2.44 (2.33, 2.56)	<0.0001	3,623	2,897 (80%)	80 (78.7, 81.3)	1.4 (1.36, 1.45)	<0.0001
2019	3,347	3,064 (91.5%)	91.5 (90.6, 92.5)	2.58 (2.47, 2.7)	<0.0001	3,333	2,880 (86.4%)	86.4 (85.3, 87.6)	1.51 (1.47, 1.56)	<0.0001
2022	2,893	2,731 (94.4%)	94.4 (93.6, 95.2)	2.66 (2.54, 2.79)	<0.0001	2,863	2,585 (90.3%)	90.3 (89.2, 91.4)	1.58 (1.53, 1.63)	<0.0001

Viral load measurements were not routinely collected in the 2012 survey round and we are therefore unable to estimate the prevalence of suppression among people living with HIV (PLHIV) for this time frame. PLHIV with missing viral load data in later survey rounds were dropped when estimating the prevalence of suppressed HIV. Estimates were generated using Poisson regression with robust standard errors with survey round as a predictor variable. Generalized estimating equations with correlation structure selection by Quasi Information Criterion value (treatment-experienced PLHIV, suppressed PLHIV: independence) were used to account for repeat participants across study rounds. 95% confidence intervals indicate the Wald confidence interval around the mean value in each category. *p*-values that coefficients are different from 0 at the level were calculated using the Wald method.

Supplementary Table 8: Prevalence of suppression among treatment-experienced RCCS participants living with HIV

		Suppressed HIV (≤1,000 copies/mL)				401-1000 copies/mL			
Survey round	*n*	Obs (%)	Prev. (95% CI)	Prev. ratio (95% CI)	*p*-value	Obs (%)	Prev. (95% CI)	Prev. ratio (95% CI)	*p*-value
2014	2,110	1,917 (90.9%)	91 (89.8, 92.1)	ref	ref	102 (4.8%)	5 (4.2, 6)	ref	ref
2015	2,821	2,580 (91.5%)	91.1 (90.1, 92.1)	1 (0.99, 1.02)	0.85	92 (3.3%)	3.3 (2.7, 4.1)	0.67 (0.52, 0.86)	0.0017
2017	3,136	2,897 (92.4%)	91.9 (91, 92.7)	1.01 (0.99, 1.02)	0.2	59 (1.9%)	1.9 (1.5, 2.5)	0.39 (0.29, 0.52)	<0.0001
2019	3,053	2,880 (94.3%)	93.6 (92.8, 94.4)	1.03 (1.01, 1.04)	0.00015	46 (1.5%)	1.6 (1.2, 2.1)	0.31 (0.22, 0.43)	<0.0001
2022	2,702	2,585 (95.7%)	95.4 (94.6, 96.1)	1.05 (1.03, 1.06)	<0.0001	36 (1.3%)	1.3 (1, 1.9)	0.27 (0.19, 0.39)	<0.0001

		201-400 copies/mL				0-200 copies/mL			
Survey round	*n*	Obs (%)	Prev. (95% CI)	Prev. ratio (95% CI)	*p*-value	Obs (%)	Prev. (95% CI)	Prev. ratio (95% CI)	*p*-value
2014	2,110	62 (2.9%)	3 (2.3, 3.8)	ref	ref	1,753 (83.1%)	83.1 (81.5, 84.7)	ref	ref
2015	2,821	37 (1.3%)	1.3 (1, 1.8)	0.45 (0.31, 0.66)	<0.0001	2,451 (86.9%)	86.9 (85.6, 88.1)	1.05 (1.02, 1.07)	<0.0001
2017	3,136	38 (1.2%)	1.2 (0.9, 1.7)	0.41 (0.28, 0.61)	<0.0001	2,800 (89.3%)	89.3 (88.2, 90.4)	1.07 (1.05, 1.1)	<0.0001
2019	3,053	36 (1.2%)	1.2 (0.9, 1.6)	0.4 (0.27, 0.6)	<0.0001	2,798 (91.6%)	91.6 (90.7, 92.6)	1.1 (1.08, 1.13)	<0.0001
2022	2,702	21 (0.8%)	0.8 (0.5, 1.2)	0.26 (0.16, 0.43)	<0.0001	2,528 (93.6%)	93.6 (92.6, 94.5)	1.13 (1.1, 1.15)	<0.0001

Viral load measurements were not routinely collected in the 2012 survey round and we are therefore unable to estimate the prevalence of suppression among treatment-experienced people living with HIV (PLHIV) for this time frame. PLHIV with missing viral load data in later survey rounds were dropped when estimating the prevalence of suppressed HIV. Estimates were generated using Poisson regression with robust standard errors with survey round as a predictor variable. Generalized estimating equations with correlation structure selection by Quasi Information Criterion value (Suppressed: AR1, 401-1000 copies/mL: exchangeable, 201-400 copies/mL: exchangeable, 0-200 copies/mL: independence) were used to account for repeat participants across study rounds. 95% confidence intervals indicate the Wald confidence interval around the mean value in each category. *p*-values that coefficients are different from 0 at the level were calculated using the Wald method.

Supplementary Table 9: List of surveyed clinics serving PLHIV who participated in the 2022 Rakai Community Cohort Study survey round.

Clinic name	2022 RCCS participants w/ HIV	
	n	%
Kasensero	660	25.6
Rakai Program Kalisizo Hub	336	13.1
Kakuuto	159	6.2
Kasasa	120	4.7
Rakai hospital	101	3.9
Taso Masaka	93	3.6
Kabira	92	3.6
Kasaali	79	3.1
Lwanda	79	3.1
Kifamba	65	2.5
Kamulegu H/C III	39	1.5
Mitukula	35	1.4
Lyantonde Hospital	25	1
Kyanamukaaka HC IV	24	0.9

Supplementary Table 10: Proportion of care seeking PLHIV prescribed DTG at surveyed clinics stratified by date and sex.

Quarter	Men			Women		
	n	DTG	% (95% CI)	n	DTG	% (95% CI)
2018 Q1	7,588	985	13 (12.2, 13.7)	13,968	2,489	17.8 (17.2, 18.5)
2018 Q2	7,879	1,142	14.5 (13.7, 15.3)	14,465	2,735	18.9 (18.3, 19.5)
2018 Q3	8,152	1,274	15.6 (14.8, 16.4)	14,821	2,860	19.3 (18.7, 19.9)
2018 Q4	8,105	2,051	25.3 (24.4, 26.3)	14,584	3,571	24.5 (23.8, 25.2)
2019 Q1	8,279	2,513	30.4 (29.4, 31.3)	15,456	3,880	25.1 (24.4, 25.8)
2019 Q2	9,398	3,323	35.4 (34.4, 36.3)	15,626	4,428	28.3 (27.6, 29)
2019 Q3	8,789	3,371	38.4 (37.3, 39.4)	15,939	5,200	32.6 (31.9, 33.4)
2019 Q4	8,773	5,683	64.8 (63.8, 65.8)	15,962	7,565	47.4 (46.6, 48.2)
2020 Q1	8,631	7,286	84.4 (83.7, 85.2)	16,423	10,611	64.6 (63.9, 65.3)
2020 Q2	8,702	7,434	85.4 (84.7, 86.2)	16,173	10,501	64.9 (64.2, 65.7)
2020 Q3	9,317	8,043	86.3 (85.6, 87)	16,408	11,156	68 (67.3, 68.7)
2020 Q4	9,724	8,746	89.9 (89.3, 90.5)	17,012	13,046	76.7 (76.1, 77.3)
2021 Q1	9,565	8,932	93.4 (92.9, 93.9)	17,561	15,347	87.4 (86.9, 87.9)
2021 Q2	10,361	9,886	95.4 (95, 95.8)	17,100	15,689	91.7 (91.3, 92.2)
2021 Q3	9,930	9,513	95.8 (95.4, 96.2)	17,803	16,748	94.1 (93.7, 94.4)
2021 Q4	9,507	9,202	96.8 (96.4, 97.1)	17,135	16,390	95.7 (95.3, 96)
2022 Q1	9,824	9,559	97.3 (97, 97.6)	17,549	17,003	96.9 (96.6, 97.1)
2022 Q2	9,667	9,431	97.6 (97.3, 97.9)	17,813	17,080	95.9 (95.6, 96.2)
2022 Q3	9,800	9,579	97.7 (97.5, 98)	17,617	17,266	98 (97.8, 98.2)
2022 Q4	9,658	9,488	98.2 (98, 98.5)	17,591	17,283	98.2 (98.1, 98.4)
2023 Q1	9,715	9,519	98 (97.7, 98.3)	17,882	17,312	96.8 (96.6, 97.1)

95% confidence intervals indicate the Wald confidence interval around the mean value in each category.

Supplementary Table 11: Proportion of Treatment-experienced viremic participant visits on which deep-sequence based prediction of drug resistance was attempted and successful.

		Treatment-experienced viremic PLHIV							
				Deep-sequence based drug resistance prediction (% of sequenced)					
		Participant-visits	Sequenced (% of par.-visits)	Any	INSTI	NNRTI	NRTI	PI	all
Overall		963	936 (97.2%)	783 (83.7%)	770 (82.3%)	677 (72.3%)	656 (70.1%)	711 (76%)	630 (67.3%)
Survey round									
	2014	193	182 (94.3%)	94 (51.6%)	90 (49.5%)	66 (36.3%)	65 (35.7%)	76 (41.8%)	57 (31.3%)
	2015	241	233 (96.7%)	215 (92.3%)	213 (91.4%)	192 (82.4%)	180 (77.3%)	190 (81.5%)	172 (73.8%)
	2017	239	235 (98.3%)	218 (92.8%)	218 (92.8%)	210 (89.4%)	207 (88.1%)	214 (91.1%)	205 (87.2%)
	2019	173	170 (98.3%)	147 (86.5%)	141 (82.9%)	119 (70%)	119 (70%)	134 (78.8%)	112 (65.9%)
	2022	117	116 (99.1%)	109 (94%)	108 (93.1%)	90 (77.6%)	85 (73.3%)	97 (83.6%)	84 (72.4%)
Sequencing approach									
	amplicon	9	9 (100%)	6 (66.7%)	5 (55.6%)	5 (55.6%)	6 (66.7%)	6 (66.7%)	4 (44.4%)
	veSeq-HIV	927	927 (100%)	777 (83.8%)	765 (82.5%)	672 (72.5%)	650 (70.1%)	705 (76.1%)	626 (67.5%)
Subtype		32	5 (15.6%)						
	A1	255	255 (100%)	218 (85.5%)	215 (84.3%)	182 (71.4%)	182 (71.4%)	195 (76.5%)	175 (68.6%)
	C	32	32 (100%)	28 (87.5%)	28 (87.5%)	23 (71.9%)	23 (71.9%)	23 (71.9%)	21 (65.6%)
	D	505	505 (100%)	426 (84.4%)	421 (83.4%)	375 (74.3%)	363 (71.9%)	395 (78.2%)	348 (68.9%)
	other	139	139 (100%)	111 (79.9%)	106 (76.3%)	97 (69.8%)	88 (63.3%)	98 (70.5%)	86 (61.9%)
	missing	32	5 (15.6%)	NA (NA%)	NA (NA%)	NA (NA%)	NA (NA%)	NA (NA%)	NA (NA%)
Viral load (log10 copies/mL)									
	(3,4]	500	484 (96.8%)	359 (74.2%)	348 (71.9%)	280 (57.9%)	257 (53.1%)	296 (61.2%)	241 (49.8%)
	(4,5]	364	357 (98.1%)	330 (92.4%)	328 (91.9%)	304 (85.2%)	305 (85.4%)	321 (89.9%)	296 (82.9%)
	(5, Inf]	99	95 (96%)	94 (98.9%)	94 (98.9%)	93 (97.9%)	94 (98.9%)	94 (98.9%)	93 (97.9%)

Supplementary Table 12: Prevalence of resistance among treatment-experienced viremic PLHIV in the Rakai Community Cohort Study, 2014 - 2022.

	Any					INSTI					NNRTI				
**Survey round**	***n***	**Obs (%)**	**Prev. (95% CI)**	**Prev. ratio (95% CI)**	***p*-value**	***n***	**Obs (%)**	**Prev. (95% CI)**	**Prev. ratio (95% CI)**	***p*-value**	***n***	**Obs (%)**	**Prev. (95% CI)**	**Prev. ratio (95% CI)**	***p*-value**
2014	62	29 (46.8%)	51.1 (40.7, 64.2)	ref	ref	90	0 (0%)	0 (NA, NA)	ref	ref	66	28 (42.4%)	46 (35.9, 58.9)	ref	ref
2015	180	95 (52.8%)	49.8 (44.2, 56.1)	0.98 (0.78, 1.22)	0.83	213	1 (0.5%)	0.4 (0.1, 3)	NA (0, Inf)		192	92 (47.9%)	46 (40.7, 52.1)	1 (0.79, 1.27)	0.99
2017	207	95 (45.9%)	44.4 (39.1, 50.4)	0.87 (0.68, 1.11)	0.26	218	2 (0.9%)	0.9 (0.2, 3.5)	NA (0, Inf)		210	91 (43.3%)	43.4 (38.3, 49.1)	0.94 (0.73, 1.22)	0.66
2019	114	50 (43.9%)	41.4 (34.2, 50.1)	0.81 (0.6, 1.09)	0.16	141	0 (0%)	0 (0, 0)	NA (0, Inf)		119	47 (39.5%)	37.6 (31.3, 45.3)	0.82 (0.61, 1.11)	0.19
2022	85	17 (20%)	27.9 (21.3, 36.5)	0.55 (0.39, 0.77)	0.00066	108	3 (2.8%)	2.7 (0.9, 8.3)	NA (0, Inf)		90	14 (15.6%)	22.2 (17.1, 28.9)	0.48 (0.34, 0.69)	<0.0001

	NRTI					PI				
**Survey round**	***n***	**Obs (%)**	**Prev. (95% CI)**	**Prev. ratio (95% CI)**	***p*-value**	***n***	**Obs (%)**	**Prev. (95% CI)**	**Prev. ratio (95% CI)**	***p*-value**
2014	65	25 (38.5%)	34.1 (25.4, 45.7)	ref	ref	76	0 (0%)	0 (0, Inf)	ref	ref
2015	180	77 (42.8%)	38.9 (33.4, 45.4)	1.14 (0.86, 1.52)	0.35	190	6 (3.2%)	3.1 (1.4, 6.6)	NA (0, Inf)	1
2017	207	72 (34.8%)	33 (28, 38.8)	0.97 (0.71, 1.33)	0.84	214	5 (2.3%)	2.2 (1, 5.3)	NA (0, Inf)	1
2019	119	29 (24.4%)	24.5 (18.3, 32.8)	0.72 (0.48, 1.08)	0.11	134	5 (3.7%)	3.8 (1.6, 9)	NA (0, Inf)	1
2022	85	6 (7.1%)	12.3 (7.7, 19.9)	0.36 (0.21, 0.63)	0.00035	97	1 (1%)	0.9 (0.1, 6)	NA (0, Inf)	1

Viral load measurements were not routinely collected in the 2012 survey round and therefore we exclude this this time frame. Estimates were generated using Poisson regression with robust standard errors with survey round as a predictor variable. Generalized estimating equations with correlation structure selection by Quasi Information Criterion value (Any, NRTI, NNRTI: AR1) were used to account for repeat participants across study rounds. 95% confidence intervals indicate the Wald confidence interval around the mean value in each category. *p*-values that coefficients are different from 0 at the level were calculated using the Wald method. INSTI = integrase strand transfer inhibitor, NNRTI = non-nucleoside reverse transcriptase inhibitor, NRTI = nucleoside reverse transcriptase inhibitor, PI = protease inhibitor.

Supplementary Table 13: Demographics of treatment-experienced viremic people living with HIV with resistance to any drug classes in the Rakai Community Cohort Study, 2014-2022.

Variable	Viremic treatment-experienced PLHIV		Risk ratio (95% CI)	*p*-value	Adj. risk ratio	Adj. *p*-value
	Genotyped	W/ resistance (%)				
**Age**						
(14,24]	98	50 (51%)	ref	ref	ref	ref
(24,34]	296	128 (43.2%)	0.81 (0.62, 1.06)	0.12	0.83 (0.64, 1.08)	0.17
(34,49]	254	108 (42.5%)	0.8 (0.6, 1.06)	0.13	0.81 (0.61, 1.08)	0.15
**Community type**						
fishing	353	147 (41.6%)	ref	ref	ref	ref
inland	295	139 (47.1%)	1.22 (0.98, 1.51)	0.07	1.23 (1, 1.51)	0.053
**Sex**						
female	346	177 (51.2%)	ref	ref	ref	ref
male	302	109 (36.1%)	0.65 (0.52, 0.82)	0.0002	0.65 (0.53, 0.81)	0.0001
**Subtype**						
A1	180	81 (45%)	ref	ref	ref	ref
C	23	6 (26.1%)	0.67 (0.36, 1.25)	0.2	0.67 (0.36, 1.24)	0.2
D	358	160 (44.7%)	0.9 (0.71, 1.12)	0.34	0.88 (0.7, 1.1)	0.26
other	87	39 (44.8%)	1.11 (0.82, 1.5)	0.49	1.06 (0.8, 1.41)	0.67

P-values are calculated from either univariate or bivariate (with survey round) Poisson regression with robust standard errors. Generalized estimating equations with correlation structure selection by Quasi Information Criterion value (age, community type, sex, subtype: AR1) were used to account for repeat participants across study rounds.

Supplementary Table 14: Prevalence of multi-class resistance among treatment-experienced viremic PLHIV in the Rakai Community Cohort Study, 2014 - 2022.

	Multi-class					Single-class				
**Survey round**	***n***	**Obs (%)**	**Prev. (95% CI)**	**Prev. ratio (95% CI)**	***p*-value**	***n***	**Obs (%)**	**Prev. (95% CI)**	**Prev. ratio (95% CI)**	***p*-value**
2014	57	23 (40.4%)	41.5 (31.2, 55.3)	ref	ref	57	1 (1.8%)	2.3 (0.4, 14.3)	ref	ref
2015	172	72 (41.9%)	38.4 (33, 44.8)	0.92 (0.7, 1.22)	0.58	172	15 (8.7%)	8.5 (5.3, 13.4)	3.69 (0.57, 23.71)	0.17
2017	205	72 (35.1%)	33.5 (28.4, 39.4)	0.81 (0.59, 1.1)	0.17	205	21 (10.2%)	10.3 (7, 15.3)	4.51 (0.7, 29.26)	0.11
2019	112	28 (25%)	25.5 (19.7, 33.1)	0.61 (0.42, 0.9)	0.013	112	20 (17.9%)	17.9 (12.1, 26.3)	7.8 (1.2, 50.68)	0.031
2022	84	5 (6%)	9.7 (6.1, 15.5)	0.23 (0.14, 0.4)	<0.0001	84	11 (13.1%)	12.9 (7.5, 21.9)	5.62 (0.83, 37.8)	0.076

Viral load measurements were not routinely collected in the 2012 survey round and therefore we exclude this this time frame. Estimates were generated using Poisson regression with robust standard errors with survey round as a predictor variable. Multi-class refers to any combination of simultaneous resistance to any of the four drug classes. Generalized estimating equations with correlation structure selection by Quasi Information Criterion value (multi-class: AR1, single-class: exchangeable) were used to account for repeat participants across study rounds. 95% confidence intervals indicate the Wald confidence interval around the mean value in each category. *p*-values that coefficients are different from 0 at the level were calculated using the Wald method.

Supplementary Table 15: Prevalence of resistance-conferring mutations among treatment-experienced viremic PLHIV in the Rakai Community Cohort Study, 2014 - 2022.

		inT97A				rtK103N				inS153Y			
**Survey round**	***n***	**Obs (%)**	**Prev. (95% CI)**	**Prev. ratio (95% CI)**	***p*-value**	**Obs (%)**	**Prev. (95% CI)**	**Prev. ratio (95% CI)**	***p*-value**	**Obs (%)**	**Prev. (95% CI)**	**Prev. ratio (95% CI)**	***p*-value**
2014	134	2 (1.5%)	8.2 (2.2, 30.6)	ref	ref	10 (7.5%)	25 (14, 44.7)	ref	ref	0 (0%)	0 (0, Inf)	ref	ref
2015	192	15 (7.8%)	9.7 (5.9, 15.7)	1.18 (0.29, 4.76)	0.82	46 (24%)	32.8 (25.7, 41.8)	1.31 (0.71, 2.42)	0.39	0 (0%)	0 (0, 0)	NA (0, Inf)	1
2017	212	21 (9.9%)	11.6 (7.7, 17.4)	1.41 (0.36, 5.57)	0.62	50 (23.6%)	27 (21.3, 34.3)	1.08 (0.58, 2.01)	0.8	0 (0%)	0 (NA, NA)	NA (0, Inf)	1
2019	159	5 (3.1%)	4.5 (1.9, 10.7)	0.55 (0.11, 2.64)	0.45	29 (18.2%)	26.3 (19.1, 36.2)	1.05 (0.54, 2.04)	0.88	0 (0%)	0 (0, 0)	NA (0, Inf)	1
2022	108	9 (8.3%)	10.2 (5.4, 19.1)	1.24 (0.29, 5.31)	0.77	8 (7.4%)	9.1 (4.6, 18)	0.37 (0.15, 0.89)	0.027	7 (6.5%)	7.8 (3.9, 15.7)	NA (0, Inf)	1

		prL23I				rtM184V				rtM41L			
**Survey round**	***n***	**Obs (%)**	**Prev. (95% CI)**	**Prev. ratio (95% CI)**	***p*-value**	**Obs (%)**	**Prev. (95% CI)**	**Prev. ratio (95% CI)**	***p*-value**	**Obs (%)**	**Prev. (95% CI)**	**Prev. ratio (95% CI)**	***p*-value**
2014	134	0 (0%)	0 (NA, NA)	ref	ref	19 (14.2%)	47.3 (33.7, 66.4)	ref	ref	5 (3.7%)	11.1 (4.6, 26.7)	ref	ref
2015	192	0 (0%)	0 (0, 0)	NA (0, Inf)	<0.0001	59 (30.7%)	42.1 (34.5, 51.4)	0.89 (0.61, 1.3)	0.55	11 (5.7%)	7.8 (4.4, 14.1)	0.71 (0.27, 1.85)	0.48
2017	212	0 (0%)	0 (0, 0)	NA (0, Inf)	<0.0001	65 (30.7%)	35 (28.7, 42.7)	0.74 (0.5, 1.09)	0.13	11 (5.2%)	5.8 (3.3, 10.4)	0.53 (0.18, 1.51)	0.23
2019	159	0 (0%)	0 (0, 0)	NA (0, Inf)	<0.0001	24 (15.1%)	22 (15.3, 31.6)	0.47 (0.28, 0.77)	0.0026	4 (2.5%)	3.7 (1.4, 10)	0.33 (0.09, 1.26)	0.11
2022	108	5 (4.6%)	6.6 (2.8, 15.2)	NA (0, Inf)	<0.0001	4 (3.7%)	5.1 (2, 13.3)	0.11 (0.04, 0.3)	<0.0001	4 (3.7%)	5 (1.9, 13)	0.45 (0.12, 1.66)	0.23

		rtP225H				rtT215Y				rtY181C			
**Survey round**	***n***	**Obs (%)**	**Prev. (95% CI)**	**Prev. ratio (95% CI)**	***p*-value**	**Obs (%)**	**Prev. (95% CI)**	**Prev. ratio (95% CI)**	***p*-value**	**Obs (%)**	**Prev. (95% CI)**	**Prev. ratio (95% CI)**	***p*-value**
2014	134	1 (0.75%)	1.92 (0.27, 13.53)	ref	ref	4 (2.99%)	11.59 (4.31, 31.19)	ref	ref	12 (8.96%)	26.59 (15.68, 45.1)	ref	ref
2015	192	6 (3.12%)	4.05 (1.84, 8.93)	2.11 (0.26, 17.23)	0.49	5 (2.6%)	4.28 (1.78, 10.28)	0.37 (0.1, 1.38)	0.14	33 (17.19 %)	24.34 (17.96, 33)	0.92 (0.51, 1.63)	0.76

2017	212	11 (5.19%)	6.14 (3.44, 10.95)	3.2 (0.42, 24.43)	0.26	5 (2.36%)	2.76 (1.16, 6.58)	0.24 (0.06, 0.89)	0.032	27 (12.74 %)	14.22 (10, 20.23)	0.53 (0.29, 1)	0.049
2019	159	9 (5.66%)	8.48 (4.5, 16)	4.42 (0.57, 34.35)	0.16	2 (1.26%)	2.04 (0.5, 8.37)	0.18 (0.03, 0.98)	0.048	10 (6.29%)	9.72 (5.34, 17.66)	0.37 (0.16, 0.81)	0.014
2022	108	3 (2.78%)	3.55 (1.15, 10.98)	1.85 (0.19, 17.55)	0.59	3 (2.78%)	3.89 (1.28, 11.83)	0.34 (0.08, 1.48)	0.15	3 (2.78%)	3.63 (1.18, 11.13)	0.14 (0.04, 0.47)	0.0016

		**inE138K**			
**Survey round**	***n***	**Obs (%)**	**Prev. (95% CI)**	**Prev. ratio (95% CI)**	***p-value***
2014	134	0 (0%)	0 (0, Inf)	ref	ref
2015	192	1 (0.52%)	0.62 (0.09, 4.41)	NA (0, Inf)	1
2017	212	0 (0%)	0 (0, 0)	NA (0, Inf)	1
2019	159	0 (0%)	0 (0, 0)	NA (0, Inf)	1
2022	108	1 (0.93%)	1.23 (0.18, 8.66)	NA (0, Inf)	1

Viral load measurements were not routinely collected in the 2012 survey round and therefore we exclude this this time frame. Estimates were generated using Poisson regression with robust standard errors with survey round as a predictor variable. Multi-class refers to any combination of simultaneous resistance to any of the four drug classes. Generalized estimating equations with independent correlation structure to aid convergence for rare mutations were used. 95% confidence intervals indicate the Wald confidence interval around the mean value in each category. *p*-values that coefficients are different from 0 at the level were calculated using the Wald method.

Supplementary Table 16: Proportion of pre-treatment viremic participant visits on which deep-sequence based prediction of drug resistance was attempted and successful.

		Pre-treatment viremic PLHIV							
		Participant-visits	Sequenced (% of par.-visits)	Deep-sequence based drug resistance prediction (% of sequenced)					
				Any	INSTI	NNRTI	NRTI	PI	all
Overall		4,981	4,924 (98.9%)	3,918 (79.6%)	2,766 (56.2%)	3,206 (65.1%)	3,176 (64.5%)	3,744 (76%)	2,461 (50%)
Survey round									
	2012	2,015	1,993 (98.9%)	1,420 (71.2%)	516 (25.9%)	979 (49.1%)	1,017 (51%)	1,387 (69.6%)	482 (24.2%)
	2014	1,249	1,228 (98.3%)	856 (69.7%)	636 (51.8%)	668 (54.4%)	659 (53.7%)	774 (63%)	502 (40.9%)
	2015	789	784 (99.4%)	757 (96.6%)	737 (94%)	719 (91.7%)	687 (87.6%)	722 (92.1%)	667 (85.1%)
	2017	487	481 (98.8%)	470 (97.7%)	465 (96.7%)	459 (95.4%)	450 (93.6%)	465 (96.7%)	450 (93.6%)
	2019	280	279 (99.6%)	264 (94.6%)	262 (93.9%)	238 (85.3%)	227 (81.4%)	253 (90.7%)	227 (81.4%)
	2022	161	159 (98.8%)	151 (95%)	150 (94.3%)	143 (89.9%)	136 (85.5%)	143 (89.9%)	133 (83.6%)
Sequencing approach									
	amplicon	2,571	2,571 (100%)	1,792 (69.7%)	666 (25.9%)	1,276 (49.6%)	1,332 (51.8%)	1,759 (68.4%)	638 (24.8%)
	veSeq-HIV	2,353	2,353 (100%)	2,126 (90.4%)	2,100 (89.2%)	1,930 (82%)	1,844 (78.4%)	1,985 (84.4%)	1,823 (77.5%)
Subtype									
	A1	1,356	1,356 (100%)	1,200 (88.5%)	851 (62.8%)	1,010 (74.5%)	1,004 (74%)	1,140 (84.1%)	758 (55.9%)
	C	149	149 (100%)	122 (81.9%)	93 (62.4%)	103 (69.1%)	104 (69.8%)	117 (78.5%)	85 (57%)
	D	1,908	1,908 (100%)	1,623 (85.1%)	1,338 (70.1%)	1,439 (75.4%)	1,400 (73.4%)	1,550 (81.2%)	1,189 (62.3%)
	other	1,504	1,504 (100%)	973 (64.7%)	484 (32.2%)	654 (43.5%)	668 (44.4%)	937 (62.3%)	429 (28.5%)
	missing	64	7 (10.9%)						
Viral load (log10 copies/mL)									
	(3,4]	1,677	1,653 (98.6%)	1,076 (65.1%)	711 (43%)	774 (46.8%)	739 (44.7%)	980 (59.3%)	551 (33.3%)
	(4,5]	1,971	1,952 (99%)	1,753 (89.8%)	1,351 (69.2%)	1,513 (77.5%)	1,505 (77.1%)	1,696 (86.9%)	1,227 (62.9%)
	(5, Inf]	541	537 (99.3%)	525 (97.8%)	469 (87.3%)	502 (93.5%)	503 (93.7%)	520 (96.8%)	460 (85.7%)
	missing	792	782 (98.7%)	564 (72.1%)	235 (30.1%)	417 (53.3%)	429 (54.9%)	548 (70.1%)	223 (28.5%)

Supplementary Table 17: Prevalence of resistance among pre-treatment PLHIV in the Rakai Community Cohort Study, 2012 - 2022.

	INSTI					NNRTI				
**Survey round**	***n***	**Obs (%)**	**Prev. (95% CI)**	**Prev. ratio (95% CI)**	***p*-value**	***n***	**Obs (%)**	**Prev. (95% CI)**	**Prev. ratio (95% CI)**	***p*-value**
2012	516	5 (1%)	1 (0.4, 2.4)	ref	ref	979	52 (5.3%)	5.4 (4.3, 6.9)	ref	ref
2014	636	12 (1.9%)	2.1 (1.2, 3.8)	2.17 (0.74, 6.33)	0.16	668	40 (6%)	6.3 (4.9, 8.1)	1.16 (0.87, 1.56)	0.31
2015	737	1 (0.1%)	0.2 (0, 1.1)	0.16 (0.02, 1.35)	0.091	719	52 (7.2%)	7.2 (5.7, 9.1)	1.32 (0.96, 1.81)	0.087
2017	465	0 (0%)	0 (0, 0)	0 (0, 0)	<0.0001	459	45 (9.8%)	9.9 (7.8, 12.4)	1.81 (1.3, 2.51)	0.0004
2019	262	0 (0%)	0 (0, 0)	0 (0, 0)	<0.0001	238	27 (11.3%)	11.5 (8.6, 15.5)	2.12 (1.45, 3.09)	0.0001
2022	150	0 (0%)	0 (0, 0)	0 (0, 0)	<0.0001	143	23 (16.1%)	14.8 (9.7, 22.7)	2.72 (1.67, 4.44)	<0.0001

	NRTI					PI				
**Survey round**	***n***	**Obs (%)**	**Prev. (95% CI)**	**Prev. ratio (95% CI)**	***p*-value**	***n***	**Obs (%)**	**Prev. (95% CI)**	**Prev. ratio (95% CI)**	***p*-value**
2012	1,017	22 (2.2%)	1.9 (1.2, 3)	ref	ref	1,387	29 (2.1%)	2.2 (1.5, 3.1)	ref	ref
2014	659	10 (1.5%)	1.7 (0.9, 3.2)	0.93 (0.44, 1.97)	0.84	774	9 (1.2%)	1.3 (0.7, 2.4)	0.6 (0.31, 1.17)	0.14
2015	687	13 (1.9%)	1.9 (1.1, 3.3)	1.03 (0.49, 2.16)	0.94	722	10 (1.4%)	1.4 (0.8, 2.5)	0.66 (0.35, 1.24)	0.19
2017	450	7 (1.6%)	1.7 (0.9, 3.2)	0.91 (0.41, 2.04)	0.82	465	8 (1.7%)	1.7 (0.8, 3.2)	0.76 (0.37, 1.58)	0.47
2019	227	4 (1.8%)	2 (1, 4)	1.05 (0.45, 2.45)	0.92	253	2 (0.8%)	0.8 (0.2, 3.4)	0.37 (0.08, 1.65)	0.19
2022	136	1 (0.7%)	0.9 (0.2, 4.3)	0.5 (0.1, 2.48)	0.4	143	1 (0.7%)	0.8 (0.1, 5.2)	0.38 (0.06, 2.47)	0.31

Estimates were generated using Poisson regression with robust standard errors with survey round as a predictor variable. Generalized estimating equations with correlation structure selection by Quasi Information Criterion value (NRTI, PI: AR1, NRTI: exchangeable) were used to account for repeat participants across study rounds. 95% confidence intervals indicate the Wald confidence interval around the mean value in each category. *p*-values that coefficients are different from 0 at the level were calculated using the Wald method. INSTI = integrase strand transfer inhibitor, NNRTI = non-nucleoside reverse transcriptase inhibitor, NRTI = nucleoside reverse transcriptase inhibitor, PI = protease inhibitor.

Supplementary Table 18: Demographics of pre-treatment people living with HIV with resistance to NNRTIs in the Rakai Community Cohort Study, 2014-2022.

Variable	Viremic treatment-experienced PLHIV		Risk ratio (95% CI)	*p*-value	Adj. risk ratio	Adj. *p*-value
	Genotyped	W/ resistance (%)				
**Age**						
(14,24]	649	53 (8.2%)	ref	ref	ref	ref
(24,34]	1603	117 (7.3%)	0.9 (0.66, 1.22)	0.5	0.92 (0.67, 1.24)	0.58
(34,49]	954	69 (7.2%)	0.94 (0.65, 1.35)	0.72	0.94 (0.65, 1.35)	0.74
**Community type**						
fishing	1529	110 (7.2%)	ref	ref	ref	ref
inland	1677	129 (7.7%)	1.13 (0.84, 1.5)	0.42	1.17 (0.88, 1.56)	0.27
**Sex**						
female	1566	135 (8.6%)	ref	ref	ref	ref
male	1640	104 (6.3%)	0.74 (0.56, 0.98)	0.035	0.72 (0.55, 0.95)	0.021
**Subtype**						
A1	1010	75 (7.4%)	ref	ref	ref	ref
C	103	8 (7.8%)	1 (0.47, 2.1)	0.99	1.01 (0.39, 2.59)	0.98
D	1439	114 (7.9%)	1.13 (0.81, 1.58)	0.46	1.12 (0.82, 1.54)	0.47
other	654	42 (6.4%)	0.9 (0.6, 1.35)	0.6	1.04 (0.72, 1.48)	0.85

P-values are calculated from either univariate or bivariate (with survey round) Poisson regression with robust standard errors. Generalized estimating equations with correlation structure selection by Quasi Information Criterion value (age, community type, sex, subtype: AR1) were used to account for repeat participants across study rounds. NNRTI = non-nucleoside reverse transcriptase inhibitor.

Supplementary Table 19: Prevalence of resistance-conferring mutations among pre-treatment viremic PLHIV in the Rakai Community Cohort Study, 2012 - 2022.

		rtK103N				inT97A				inS153Y			
**Survey round**	***n***	**Obs (%)**	**Prev. (95% CI)**	**Prev. ratio (95% CI)**	***p*-value**	**Obs (%)**	**Prev. (95% CI)**	**Prev. ratio (95% CI)**	***p*-value**	**Obs (%)**	**Prev. (95% CI)**	**Prev. ratio (95% CI)**	***p*-value**
2012	1,223	5 (0.4%)	1.9 (0.8, 4.5)	ref	ref	17 (1.4%)	6.4 (4, 10.2)	ref	ref	0 (0%)	0 (NA, NA)	ref	ref
2014	1,308	15 (1.1%)	2.9 (1.7, 4.8)	1.56 (0.59, 4.13)	0.37	49 (3.7%)	9.6 (7.3, 12.6)	1.49 (0.89, 2.48)	0.13	0 (0%)	0 (0, 0)	NA (0, Inf)	<0.0001
2015	838	27 (3.2%)	3.9 (2.7, 5.7)	2.1 (0.81, 5.42)	0.13	65 (7.8%)	9.3 (7.4, 11.7)	1.45 (0.87, 2.43)	0.16	0 (0%)	0 (0, 0)	NA (0, Inf)	<0.0001
2017	514	33 (6.4%)	7 (5, 9.7)	3.74 (1.47, 9.52)	0.0056	48 (9.3%)	10.2 (7.8, 13.3)	1.59 (0.93, 2.72)	0.091	0 (0%)	0 (0, 0)	NA (0, Inf)	<0.0001
2019	294	17 (5.8%)	7.5 (4.7, 12)	4.04 (1.5, 10.85)	0.0056	33 (11.2%)	13.7 (10, 18.9)	2.14 (1.22, 3.76)	0.0083	0 (0%)	0 (0, 0)	NA (0, Inf)	<0.0001
2022	170	14 (8.2%)	10.2 (6.2, 17)	5.49 (2, 15.06)	0.00095	10 (5.9%)	7 (3.8, 12.8)	1.09 (0.51, 2.34)	0.83	10 (5.9%)	7.8 (4.3, 14.3)	NA (0, Inf)	<0.0001

		rtE138A				prL23I				prL33F			
**Survey round**	***n***	**Obs (%)**	**Prev. (95% CI)**	**Prev. ratio (95% CI)**	***p*-value**	**Obs (%)**	**Prev. (95% CI)**	**Prev. ratio (95% CI)**	***p*-value**	**Obs (%)**	**Prev. (95% CI)**	**Prev. ratio (95% CI)**	***p*-value**
2012	1,223	5 (0.4%)	1.9 (0.8, 4.5)	ref	ref	0 (0%)	0 (0, Inf)	ref	ref	3 (0.2%)	1.2 (0.4, 3.6)	ref	ref
2014	1,308	18 (1.4%)	3.3 (2.1, 5.3)	1.75 (0.73, 4.21)	0.21	1 (0.1%)	0.2 (0, 1.3)	NA (0, Inf)	1	7 (0.5%)	1.4 (0.7, 3.1)	1.24 (0.37, 4.17)	0.73
2015	838	26 (3.1%)	3.7 (2.5, 5.4)	1.97 (0.79, 4.94)	0.15	0 (0%)	0 (0, 0)	NA (0, Inf)	1	7 (0.8%)	1 (0.5, 2.1)	0.86 (0.22, 3.32)	0.82
2017	514	12 (2.3%)	2.5 (1.4, 4.4)	1.35 (0.48, 3.81)	0.57	0 (0%)	0 (0, 0)	NA (0, Inf)	1	8 (1.6%)	1.7 (0.9, 3.4)	1.49 (0.4, 5.64)	0.55
2019	294	10 (3.4%)	4.2 (2.3, 7.7)	2.24 (0.77, 6.52)	0.14	0 (0%)	0 (0, 0)	NA (0, Inf)	1	5 (1.7%)	2 (0.9, 4.9)	1.76 (0.42, 7.37)	0.44
2022	170	7 (4.1%)	5.4 (2.6, 11.1)	2.86 (0.92, 8.92)	0.07	6 (3.5%)	4.9 (2.2, 10.6)	NA (0, Inf)	1	5 (2.9%)	3.5 (1.5, 8.3)	3.04 (0.73, 12.68)	0.13

		prG73V				rtF77L				rtG190A			
**Survey round**	***n***	**Obs (%)**	**Prev. (95% CI)**	**Prev. ratio (95% CI)**	***p*-value**	**Obs (%)**	**Prev. (95% CI)**	**Prev. ratio (95% CI)**	***p*-value**	**Obs (%)**	**Prev. (95% CI)**	**Prev. ratio (95% CI)**	***p*-value**

2012	1223	0 (0%)	0 (NA, NA)	ref	ref	0 (0%)	0 (NA, NA)	ref	ref	2 (0.16%)	0.87 (0.22, 3.44)	ref	ref
2014	1308	0 (0%)	0 (0, 0)	NA (0, Inf)	<0.0001	0 (0%)	0 (0, 0)	NA (0, Inf)	<0.0001	5 (0.38%)	1.04 (0.43, 2.51)	1.2 (0.3, 4.8)	0.8
2015	838	0 (0%)	0 (0, 0)	NA (0, Inf)	<0.0001	0 (0%)	0 (0, 0)	NA (0, Inf)	<0.0001	6 (0.72%)	0.85 (0.38, 1.88)	0.98 (0.2, 4.82)	0.98
2017	514	0 (0%)	0 (0, 0)	NA (0, Inf)	<0.0001	0 (0%)	0 (0, 0)	NA (0, Inf)	<0.0001	4 (0.78%)	0.85 (0.32, 2.27)	0.99 (0.18, 5.34)	0.99
2019	294	0 (0%)	0 (0, 0)	NA (0, Inf)	<0.0001	0 (0%)	0 (0, 0)	NA (0, Inf)	<0.0001	3 (1.02%)	1.27 (0.41, 3.91)	1.46 (0.25, 8.69)	0.67
2022	170	5 (2.94%)	4.13 (1.71, 10.02)	NA (0, Inf)	<0.0001	3 (1.76%)	2.72 (0.87, 8.53)	NA (0, Inf)	<0.0001	3 (1.76%)	2.37 (0.77, 7.32)	2.74 (0.46, 16.26)	0.27

		**rtM41L**			
**Survey round**	***n***	**Obs (%)**	**Prev. (95% CI)**	**Prev. ratio (95% CI)**	***p*-value**
2012	1223	4 (0.33%)	1.52 (0.57, 4.05)	ref	ref
2014	1308	5 (0.38%)	1.01 (0.41, 2.48)	0.67 (0.19, 2.29)	0.52
2015	838	16 (1.91%)	2.3 (1.42, 3.74)	1.52 (0.51, 4.52)	0.46
2017	514	10 (1.95%)	2.16 (1.17, 3.99)	1.42 (0.45, 4.52)	0.55
2019	294	8 (2.72%)	3.4 (1.72, 6.74)	2.24 (0.68, 7.4)	0.18
2022	170	3 (1.76%)	2.48 (0.8, 7.7)	1.63 (0.36, 7.3)	0.52

Estimates were generated using Poisson regression with robust standard errors with survey round as a predictor variable. Multi-class refers to any combination of simultaneous resistance to any of the four drug classes. Generalized estimating equations with independent correlation structure to aid convergence for rare mutations were used. 95% confidence intervals indicate the Wald confidence interval around the mean value in each category. *p*-values that coefficients are different from 0 at the level were calculated using the Wald method.
